# Ppe.CR.1 DNA test for predicting chilling requirement in peach

**DOI:** 10.1038/s41598-023-27475-w

**Published:** 2023-01-18

**Authors:** Gizem Demirel, Alejandro Calle, John Mark Lawton, Omer Atagul, Wanfang Fu, Ksenija Gasic

**Affiliations:** 1 Department of Plant and Environmental Sciences, Clemson University, Clemson, SC 29634 USA; 2 Department of Breeding and Genetics, Fruit Research Institute, 32500 Isparta, Turkey; 3 East Mediterranean Transitional Zone Agricultural Research Institute, 46060 Kahramanmaras, Turkey

**Keywords:** Genetic markers, Plant breeding

## Abstract

Chilling requirement (CR) is an important agronomic trait controlling the floral bud break for proper flowering in peach. Even though it has been widely researched and several peach CR quantitative trait loci (QTLs) have been identified, no diagnostic DNA tests validated in the U.S. peach breeding germplasm are available for this trait. Breeders and growers need a simple DNA test to predict the CR of peach cultivars for their particular environment. Therefore, we developed a quick and reliable Kompetitive Allele Specific PCR (KASP) DNA test using haplotype information from 9K IPSC genotype data of the U.S. peach germplasm integrating four CR-associated SNP markers from the previously reported CR QTL region on linkage group 1. Four KASP assays (Ppe.CR.1-1 to -4) were developed and validated on 77 peach cultivars, and nine accessions from two F_2_ populations, with 96 and 74% accuracy in determining expected CR genotype (compared to SNP array) and predicting phenotype, respectively. Furthermore, the Ppe.CR.1 showed 80% accuracy in predicting the precise CR phenotype in the Clemson University peach breeding material. Only one Ppe.CR.1 KASP assay (Ppe.CR.1-1) is needed to distinguish between haplotypes with CR lower and higher than 800 chilling hours, and two Ppe.CR.1 assays (Pp.CR.1-1 and -4), are capable of distinguishing low, moderate, and high CR alleles. Coupled with the crude DNA extraction, the Ppe.CR.1 DNA test provides a low-cost option for breeders and growers to predict CR in peach material with more than 70% accuracy.

## Introduction

Peach [*Prunus persica* (L.) Batsch] is the third most economically relevant temperate fruit tree crop worldwide, second only to apples and pears, with a global annual production of ~ 25 million tons^1^. Peaches are primarily grown in temperate zones, between latitudes 30 and 45° N and S^2^, and their production requires enough winter chill to ensure proper flowering and fruit set. Chilling requirement (CR) is an important factor in plant growth and development. It refers to the minimum number of hours of cool temperatures that a plant needs to undergo in order to break dormancy and begin to grow in the spring. Different peach cultivars have different chilling requirements, which can range from as low as 0 h to as high as 1500 h or more. Therefore, breeding for chilling requirement can be an important consideration in the development of new cultivars, as cultivar adaptation to the specific climatic condition of the growing area is essential to ensure peach production, avoiding flowering under non-optimal climatic conditions. Additionally, this trait has gained a popularity in recent decades^3,4^_,_ as global warming is resulting in warmer winters, with insufficient chilling and early bloom, which is threatening peach production^5^. Higher winter temperatures reduce the number of chill hours (CH) available affecting dormancy release, and as a consequence reducing yield and fruit quality of temperate fruit tree species^6,7^. Insufficient chill accumulation has become more frequent in the Southeast U.S. affecting the sustainability of peach production^8^. Under this warmer winter scenario, low chill cultivars may have regular production, but high chill cultivars have difficulties blooming and even leafing out displaying irregular floral and leaf bud break, delayed bud break, and low fruit set and yield^9,10^. On the other hand, due to early fulfillment of CR and blooming low chill cultivars are in danger from frost damage^11,12^. Thus, breeding for targeted CR and heat requirements^13^ has gained momentum as impacts from climate change threaten the sustainability of temperate fruit production through the lack of chilling and/or abnormal temperatures during bloom.

Previous studies have highlighted the physiology and the genetics of CR and bloom date (BD) in the species and identified regions in the peach genome associated with these traits^14–19^. One of the first mapping studies for CR and BD in peach^14^, using a simple sequence repeat (SSR) linkage map of an F_2_ population of ‘Contender’ × ‘Fla.92-2C’ [1050 and 300 chilling hours (CH) respectively], reported 20 quantitative trait loci (QTLs) associated with CR and BD. The most significant QTLs for CR and BD were reported on linkage groups (LGs) 1 (Chr1: 43.47-43.68 Mbps; peach genome v2.a.01^20^) and 7 (Chr7: 14.89–18.03 Mbps) in the two and four years that CR and BD were evaluated in this material, respectively. In a follow-up study, contrasting F_2_ individuals (ranging from 300 to 1000 CR) derived from the ‘Contender’ × ‘Fla-92-2C’ family were used to reconstruct low- and high-chilling haplotypes within the three most significant QTLs on LGs 1 (Chr1: 42.89–43.47 Mbps), 4 (Chr4: 12.52–14.14 Mbps), and 7 (Chr7: 15.62–18.24 Mbps), identified in four different seasons (2006–2009), using whole genome resequencing^16^. This study detected single nucleotide polymorphic sites (SNPs), small deletions and insertions, and large structural variants associated with low-chill haplotypes. It characterized the candidate genes associated with CR, confirming that the most important CR region in the peach genome is at the bottom of LG1 explaining up to 44% of the phenotypic variation. In parallel, Romeu et al.^15^ identified a major QTL, stable across two years, associated with CR and BD at the bottom of LG1 (Chr1: 35.51–47.47 Mbps) in the ‘V6’ (600 CH) map constructed with SNP markers from the IPSC peach 9K Infinium® II array^21^. Soon after, Bielenberg et al.^17^ used SSRs combined with SNPs generated via the genotyping-by-sequencing (GBS) approach^22^ to construct the ‘Hakuho’ × ‘UFGold’ (900 and 400 CH, respectively) F_2_ linkage map and mapped 10 and 19 QTLs associated with CR and BD, respectively. The most significant QTLs across phenotyped years (two for CR and seven for BD) were reported on LGs 1 (Chr1: 42.80–44.32 Mbps) and 4 (Chr4: 10.26–14.74 Mbps) for CR, and LGs 1 (Chr1: 37.71–43.09 Mbps) and 7 (Chr7: 15.70–19.87 Mbps) for BD. Recently, Cantín et al.^18^, using an almond × peach F_2_ linkage map derived from a cross between ‘Texas’ (high CH cultivar) and ‘Earlygold’ (low CH cultivar)^23^, mapped the most significant BD QTLs on LG1 (Chr1: 1.70–10.52 Mbps) and CR QTLs on LG6 (Chr6: 0.45–4.02 Mbps) over two consecutive years. The latest attempt to unravel the important regions associated with productivity traits in the peach genome via pedigree-connected peach germplasm and the 9K SNP Illumina array^21^ confirmed BD QTLs on LGs 1 (Chr1: 43.06–45.59 Mbps), 4 (Chr4: 11.96–12.97 Mbps), and 7 (Chr7: 15.51–17.23Mbps)^19^ collected at two different locations over two years. Thus, three major QTL regions on LGs 1, 4, and 7 in the peach genome, essential for controlling CR and BD, emerged from these studies. Especially dominant is the bottom region of LG1, where major CR and BD QTLs were detected in several peach mapping populations. This QTL region of LG1 spanned from 34.8 to 47.4 Mbps of chromosome (Chr) 1 (peach genome v2.a.01^20^) with an overlapping region for all detected QTLs within studies between 42.5 to 44.0 Mbps. In this region, a tandem repeat of six *MADS-box* genes (Chr1: 43.41–43.48 Mbps), named *dormancy-associated MADS-box* (*PpeDAM* genes), were identified^24^. Two dormancy-associated genes (*PpeDAM5* and *PpeDAM6*) were identified in this region as the most robust candidate genes for the major QTL signal at the lower end of the chromosome 1^16^.

Despite all efforts to understand the genetic control of CR in peach and its importance for the geographic distribution of cultivation, there are no widespread molecular tools available to breeders for early prediction of these traits validated in large peach germplasm. Since CR and BD can only be identified after plants reach reproductive maturity and require multiple years of observation, the best way to improve breeding efficiency and increase the chance of developing peach cultivars adapted to future climatic conditions is to use DNA information in the breeding process. Marker-assisted selection allows parental and seedling selection early in the plant development cycle and is essential in breeding traits like CR. Thus, the vast genetic resources available for the species^25–27^, such as the peach reference genome^20,28^, SNP arrays^21,29^, and application of the GBS^17^ have opened up possibilities to develop user-friendly and cost-effective molecular tools for marker-assisted selection. More recently, Kompetitive Allele-Specific PCR (KASP) assays have emerged as the method of choice for developing SNP-specific DNA tests. KASP uses three assay-specific non-labeled oligos: two forward primers in the same reaction, which are complementary to one of the SNPs in alleles at the trait locus, and one common reverse primer. Each forward primer harbors a unique tail sequence that corresponds with a universal fluorescence resonance energy transfer (FRET), one labeled with FAM dye and the other with HEX for signal generation^30,31^. Recently, the KASP technique has gained popularity because it can rapidly, cost-effectively, and reliably determine SNP genotypes^31–33^.

To date, only one attempt to establish a DNA-based toolkit for predicting CR trait has been reported^34^. A high-resolution melting (HRM) analysis-based genotyping tool kit was developed based on CR-associated QTLs reported in previous studies^14–17^. However, in validating this toolkit, the authors used only 27 peach cultivars, mainly from Taiwan and Southeast Asia. Furthermore, insufficient information on these DNA assays was published, so the study could not be replicated in different germplasm. Recently, Demirel et al.^35^ identified alleles associated with low, moderate, and high CR in the three major regions on Chrs 1, 4, and 7 in the peach genome, laying the foundation for the development of DNA test(s) for predicting CR. Therefore, the objective of this study was to develop a rapid and unambiguous DNA test for major CR associated region on Chr1^35^, using KASP technology, and validate it in the U.S. peach breeding relevant germplasm.

## Methods

### Plant material

Eighty peach cultivars from the U.S. peach germplasm and nine individuals from two F_2_ populations, ‘Contender’ × ‘Fla.92-C’ (designated as A-pop), and ‘Hakuho’ × ‘UFGold’ (designated as C-pop), representing CR requirement from 150 to 1300 CH were used in development and validation of the Ppe.CR.1 DNA assays (Table 1). For 41 samples included in the study, SNP genotyping was previously obtained using the peach 9K SNP array^21^, and CR alleles were known^35^. In addition, 161 selections from the Clemson University Peach Breeding (CUPB) program and 25 commercial peach cultivars were used to test the KASP assay’s performance using a rapid DNA extraction^36^. All the plant material is maintained at Clemson University Musser Fruit Research Center in Seneca, SC (Latitude: 34.639038, Longitude: -82.935244, Altitude: 210 MSL), under standard commercial practices for the region.

**Table 1:** Chilling requirement (CR) of the U.S. peach breeding germplasm and selections, and Ppe.CR.1 KASP assay prediction of CR in peach germplasm and comparison with 9K IPSC SNP array data (www.rosaceae.org, tfGDR1048/b).

Name	Type	Ppe.CR.1		Actual CR phenotype	KASP predicted phenotype
		KASP	Array	(CH)	(CH)
Panamint	Nectarine	H | H		150	> 850
Tropic Prince	Peach	L | L		150	< 350
TexFirst	Peach	L | L		175	< 350
Diamante	Peach	L | L-M		294	< 450
A007	Peach	M | M		350	< 450
A009	Peach	M | M		350	650–850
A066	Peach	M | M		350	650–850
A130	Peach	M | M		350	650–850
A151	Peach	M | M		350	650–850
A209	Peach	M | M	M | M	350	650–850
C005	Peach	L | L		350	< 350
C008	Peach	L | L		350	< 350
C045	Peach	L | L		350	< 350
Desert Gold	Peach	L | L		350	< 350
**Peento**	Peach	**M | M**	**L | M**	450	650–850
Sunlite	Nectarine	H | M		450	> 750
Galaxy	Peach	M | M	M | M	550	650–850
Bradley	Nectarine	M | M	M | M	650	650–850
Empress	Peach	M | L-M		650	300–700
Fairlane	Nectarine	M | M		650	650–850
LeGrand	Nectarine	M | M		650	650–850
Springprince	Peach	M | L-M	M | L-M	650	400–700
Springtime	Peach	H | L-M	H | L-M	650	500–800
Suncrest	Peach	H | M		650	> 750
Kakamas	Peach	L-M | L-M		675	300–700
Coronet	Peach	M | L-M		700	400–700
Crimson Lady	Peach	M | L-M	M | L-M	700	400–700
Fortyniner	Peach	H | H		700	> 850
**Saturn**	Peach	**M | M**	**L | M**	700	650–850
Stark Saturn	Peach	H | M		700	> 750
Caroking	Peach	M | M	M | M	750	650–850
Chinese Cling	Peach	M | M	M | M	750	650–850
Dwarf Elberta	Peach	H | M		750	> 750
**Fantasia**	Nectarine	**M | M**	**H | M**	750	650–850
Federica	Peach	M | M		750	650–850
Flavorcrest	Peach	M | M		750	650–850
Flavortop	Nectarine	H | M		750	> 750
Harvester	Peach	M | M	M | M	750	650–850
Hiley	Peach	H | H	H | H	750	> 850
Slappey	Peach	H | M	H | M	750	> 750
White Hale	Peach	M | M		750	650–850
Westbrook	Nectarine	M | M	M | M	750	650–850
Joanna Sweet	Peach	M | M		800	650–850
Loring	Peach	M | M	M | M	800	650–850
O'Henry	Peach	H | M	H | M	800	> 800
Orange Cling	Peach	H | H	H | H	800	> 850
Cumberland	Peach	M | M	M | M	850	650–850
Fayette	Peach	M | M	M | M	850	650–850
Harblaze	Nectarine	H | M		850	> 800
JH Hale	Peach	M | M	M | M	850	650–850
Julyprince	Peach	H | M	H | M	850	650–850
Red Baron	Peach	H | M		850	> 850
Redglobe	Peach	H | M	H | M	850	> 800
Redtop	Peach	H | M		850	> 800
Rio Oso Gem	Peach	H | M	H | M	850	> 800
Somervee	Peach	H | M		850	> 800
Summerprince	Peach	M | M	M | M	850	650–850
Vinegold	Peach	M | M		850	650–850
Hakuho	Peach	H | M	H |M	900	> 800
September Snow	Peach	H | L-M		900	500–800
Biscoe	Peach	M | M	M | M	925	650–850
Candor	Peach	H | H	H | H	950	> 850
Cardinal	Peach	M | M		950	650–850
Champion	Peach	H | H		950	> 850
Cresthaven	Peach	H | M		950	> 800
Dixired	Peach	H | M		950	> 800
Early Crawford	Peach	H | H	H | H	950	> 850
Harrow Diamond	Peach	H | M		950	> 800
Marigold	Peach	M | M		950	650–850
Morton	Nectarine	H | H		950	> 850
Redhaven	Peach	H | M	H | M	950	> 800
Vivid	Peach	M | M		950	650–850
White Heath Cling	Peach	H | H		950	> 850
Admiral Dewey	Peach	H | H	H | H	1000	> 850
Greensboro	Peach	M | M	M | M	1000	650–850
Intrepid	Peach	H | M	H | M	1050	> 800
Nectar	Peach	H | H		1050	> 850
Salway	Peach	H | H		1050	> 850
China Pearl	Peach	H | M	H | M	1100	> 850
Polly	Peach	H | M		1100	> 800
Mountain Rose	Peach	H | M		1150	> 800
Yumyeong	Peach	M | M	M | M	1200	650–850
Amarillo Tardio	Peach	H | H		1300	> 850

Bold accessions indicate disagreement between KASP and array data. Predicted CR for Ppe.CR alleles are indicated in the table: low (L), low-moderate (L-M), moderate (M), and high (H). *Chill hour (CH) data obtained from the literature.

### Chilling requirement phenotyping

The plant material used in developing and validating the Ppe.CR.1 DNA assays were primarily selected to ensure a broad representation of CR data. CR of this material was obtained from the literature^4,14,17,37^. Furthermore, CR for 186 cultivars (161 selections from CUPB program and 25 commercial peach cultivars) was obtained in the greenhouse in the 2021/2022 season by budbreak forcing. Hourly average temperatures were recorded from the weather station at Musser Fruit Research Center from October 15th until March 15th to calculate the sum of chill hours (CHs) using Weinberger^38^ model (CH = ∑CH {Tt > 7.2 °C, CHs = 0, 0 < Tt ≤ 7.2 °C, CHs = 1). Three terminal shoots (~ 35 cm in length) were collected for each genotype every 100 CH starting at 200 CH until 1000 CH. Shoots were placed in Oasis® floral foam micro bricks and forced in a greenhouse under 16 h of photoperiod at 25 °C. Bud break progression was evaluated after 14 days, and CR was considered fulfilled when 50% of floral buds opened.

Peach germplasm and selections were arbitrarily grouped into four phenotypic CR classes: low (< 400), low-moderate (400–650), moderate (650–850), and high CR (≥ 850).

### DNA extraction

Two DNA extraction methods have been utilized. High DNA quality was extracted from young leaf tissues of cultivars and accessions used in validation (Table 1) following the modified method from Edge-Garza et al.^39^. The DNA concentration for each sample was checked with a Thermo Scientific™ Nanodrop™ 8000 spectrophotometer and analyzed on 2% agarose electrophoresis gel. DNA was diluted in nuclease-free water to achieve the recommended concentration for KASP assays of 2.5 ng/μL.

A rapid crude DNA extraction method was used to extract DNA from 161 advanced selections from the CUPB program (Supplementary Table S1), following the protocol described by Noh et al.^36^. One 3 mm leaf disc in diameter per sample was placed in a 96-well plate well and 50 µL/well of freshly prepared Buffer A (100 mM NaOH, 2% Tween 20). The plate was centrifuged for 2 min at 2500 rpm to ensure leaf disc immersion in the buffer, heated for 10 min at 95 °C, and 50 µL/well of Buffer B (100 mM Tris–HCl pH 8, 2 mM EDTA) was added, and gently mixed by inversion. Then, the plate was centrifuged at 2500 rpm for 2 min and kept at 4 °C overnight. The following day, 100 µL/well of DNase/RNase-free water was added, and the DNA concentration range in each plate well was checked with a Nanodrop spectrophotometer. DNA concentration for crude extractions ranged from 100 to 250 ng/µL, and DNA quality ratios (absorbance at 260/280 and 260/230) were close to two. The plate was diluted with DNase/RNase-free water until the recommended concentration of 5 ng/µL was reached.

### Primer design

The region on LG1 where major CR and BD QTLs, previously mapped in several peach populations^16–19^, overlap was considered for marker development. Haplotype/allele effects, obtained from Demirel et al.^35^, were determined as the mean phenotypic value of all individuals with the presence of a particular haplotype/allele. Twelve unique haplotypes (alleles) on Chr1, identified within previously published CR and BD QTL region (Supplementary Fig. S1), could be distinguished with five SNPs (SNP_IGA_134730, SNP_1_46157131, SNP_IGA_134631, SNP_IGA_134518 and SNP_IGA_134484; Supplementary Table S7)^35^. Thus, five SNPs that distinguished twelve unique haplotypes on this region of Chr1 between 43.58 and 43.78 Mbp^35^ were chosen for KASP assay development. However, out of five SNPs in the haploblock, SNP_IGA_134518 was not suitable for the KASP assay. Removal of this marker from the haploblock collapsed the number of haplotypes from 12 to seven (Table 2; Supplementary Table S7).

**Table 2:** Ppe.CR.1 allele information and their associated phenotypes determined in 86 accessions from Demirel et al.^35^.

Haplotype region (Mbp)	SNP	Assay	Allele | phenotypic effect						
			3|L	4|L-M	5|M	1|H	6|H	2|H	7|H
Chr1: 43.58–43.78	SNP_IGA_134730	Ppe.CR.1–1	B	B	B	A	A	A	A
	SNP_1_46157131	Ppe.CR.1–2	B	B	B	A	B	A	B
	SNP_IGA_134631	Ppe.CR.1–3	A	B	A	A	A	A	A
	SNP_IGA_134484	Ppe.CR.1–4	A	B	B	A	A	B	B
N			7	25	61	13	17	27	14
Chilling hours			291	604	772	855	860	869	903

The number of individuals (N) with the presence of allele, and mean phenotype effect (chilling hours) for each allele are indicated.

*Chr* chromosome, *L* low, *M* moderate, *H* high chill requirement.

Primers were designed for each of the four SNPs following Fleming et al.^33^ protocol. In short, for each specific KASP assay, two forward primers (one for each SNP allele) and one common reversed primer were manually designed in Geneious 11.1.5 (Biomatters Ltd, Auckland NZ) software. ‘A’ allele from the SNP array (A or T nucleotide) was labeled with FAM fluorescence, and ‘B’ allele (G or C nucleotide) with HEX fluorescence. Primer design criteria were as follows: GC content between 30 and 55%; annealing temperature (Tm) of 62–66 °C; 21–30 bp length; product size of 42–100 bp; secondary structure more positive than −9 kcal/mole; no more than four di-nucleotides; no more than 4 or 5 identical nucleotides in a row; and no more than 3 Gs and/or Cs in the last 5 bp of the primer. Primers were validated in silico with the Primer-BLAST tool against the NCBI database. FAM tag was added at the *5’* end of the forward primer ending with SNP A (nucleotide A or T), and a HEX tag at the *5’* end of the forward primer ending with SNP B (nucleotide C or G).

### KASP assay

The reaction mixture and PCR conditions followed the protocol described by Fleming et al.^33^. In short, a marker-specific primer mix was created by adding 12 μM each of allele-specific forward primers, 30 μM reverse primer, and 4.6 mM Tris–HCl (pH 8.0). The reaction mixture for each assay was created using KASP reagent 2 × PACE™ 2.0 genotyping master mix following manufacturer suggestions (3CR Bioscience, Harlow, Essex, UK). A total reaction volume of 10 μL contained 5 μL of DNA (2.5 ng/μL) or water (non-template control) in a final concentration of 1 × PACE™ 2.0 genotyping master mix with no ROX dye and 0.100–0.200 μM of each marker specific primer. The plate was set up as follows: three replicates of no-template controls (NTCs) and each positive control (DNA samples of known genotype) for AA, AB, and BB genotypes were used in row A of the 96 well-plate (Supplementary Table S2). The reminding wells were filled with unreplicated samples of unknown genotypes. Positive controls for each assay were chosen from plant material previously genotyped with the IPSC 9K peach SNP array v1^21^.

Amplification in a Bio-Rad CFX Connect Real-Time PCR Detection System was performed for the validation plate following standard protocol: 15 min at 94 °C, a touchdown phase of 10 cycles at 94 °C for 20 s, and at 61 °C for 60 s with a 0.6 °C decrease in temperature per cycle, followed by 40 cycles of 94 °C for 20 s, 55 °C for 60 s, and 23 °C for 30 s (for accurate plate reading). End-point PCR reactions were conducted on Bio-Rad T100 thermal cyclers using crude DNA extracts of the 161 CUPB advanced selections, following the same protocol excluding the reading step (23 °C for 30 s), for 25 cycles, and FRET signals were read on the Bio-Rad CFX Connect Real-Time PCR Detection System.

### Data analysis

Cycle 25 of real-time PCR was selected for the following analyses for each KASP assay to maximize separation between genotype clusters and minimize background amplification. The relative fluorescence units (RFU) values of this cycle were transferred to the template developed by Fleming et al.^33^, and the proposed protocol was followed to determine the final genotype of each sample.

A template spreadsheet designed by Fleming et al.^33^ assigns genotypes automatically based on default or user-specified parameters (Supplementary Tables S3–S6). In this spreadsheet, delta value thresholds can be manually adjusted so that automatic genotype assignments correspond to what is seen in the scatterplot of relative FAM and HEX fluorescence values. In this study, no-template control thresholds were adjusted as the default threshold of 20 for both FAM and HEX for all the SNPs was adequate (Supplementary Tables S3–S6). The threshold for heterozygous signals (lower/upper bounds) in RT-PCRs was adjusted as follows: -10/34 for Ppe.CR.1-1; -40/10 for Ppe.CR.1-2; -15/40 for Ppe.CR.1-3; and -18/20 for Ppe.CR.1-4 (Supplementary Tables S3–S6).

### Experiments involving plants

Relevant guidelines and regulations have been followed for all experiments involving plants.

## Results

### Chilling requirement phenotyping

The CR of the U.S. peach breeding germplasm ranged from 150 (‘Panamint’ and ‘Tropic Prince’) to 1300 CH (‘Amarillo Tardío’; Table 1), with only 16 accessions needing less than 450 CH to reach bloom. Eight accessions had low to moderate CR (450–650 CH), and most of the accessions had moderate (650–850 CH; N = 35) to high CR (≥ 850 CH; N = 25) (Table 1).

For the CUPB material, CR ranged from 300 to more than 900 CHs (Supplementary Table S1). Clear skewed segregation from medium to high CR was observed in this material, with only eight genotypes having less than 450 CH, 37 having between 450 and 650 CH, 48 having between 650 and 850 CH, and the remaining (68) having 850 or more CH (Supplementary Table S1).

### KASP genotyping

Seven unique haplotypes (alleles) on Chr1 could be identified with the developed KASP assays (Table 2; Supplementary Table S7). Mean phenotypic values of these haplotypes in the U.S peach breeding germplasm ranged from 291 to 903 CH (Table 2). Out of all seven alleles, one was associated with low (L) CR (≤ 450 CH; allele 3), one with low-moderate (L-M) CR (450–650 CH, allele 4), one with moderate (M) CR (650–850 CH; allele 5), and the four remaining alleles with high (H) CR (≥ 850 CH; alleles 1, 2, 6, and 7) (Table 2).

**Figure 1:**
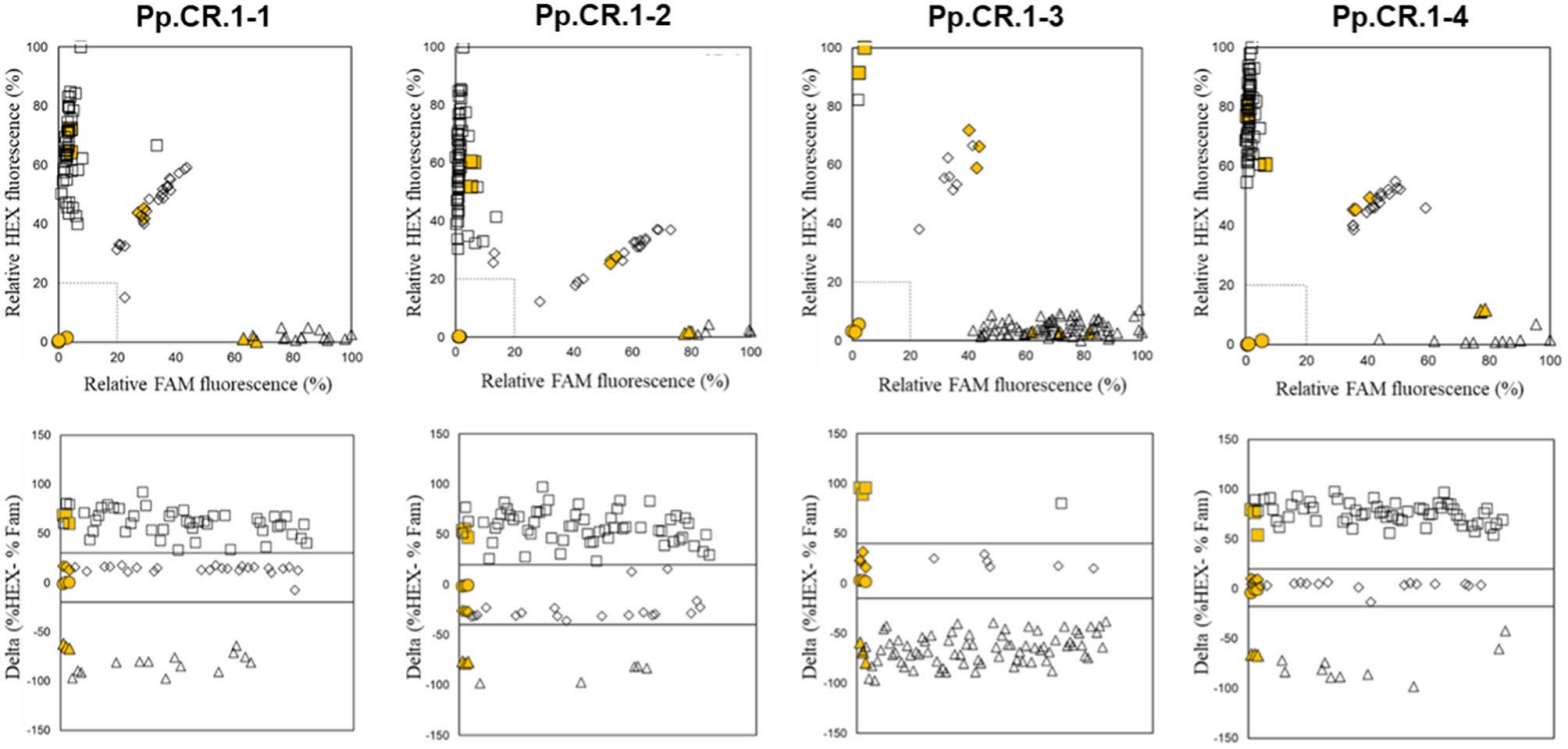
Validation of the Ppe.CR.1 KASP assays in the U.S. peach breeding germplasm. Yellow shapes show positive controls, empty shapes show unknowns, with the shape indicating the assigned genotype as follows: circle = no template/no amplification, triangle = AA, diamond = AB, square = BB. Dashed lines in plots in the top row demonstrate bounds for no amplification (% fluorescence <  ~ 20% for both fluorophores). Dashed lines in plots in the bottom row indicate delta value.

The KASP assays (Table 3) designed to identify the genotypes for each of the four SNPs successfully amplified and distinguished between genotypes (AA, AB, and BB) (Supplementary Tables S3–S6). Four clusters, indicating genotypes and NTCs, were observed for each KASP assay (Fig. 1), all successful in genotyping and haplotype assignment of the 84 peach samples. The KASP genotypes of samples for which SNP genotyping data were available (N = 36) matched for all samples except ‘Fantasia’ and ‘Saturn’ for Ppe.CR.1-1 and -4 and ‘Peento’ for Ppe.CR.1-4, providing 96.6% accuracy in assigning correct genotype when comparing with 9K SNP array data (Supplementary Table S8). For these three cultivars, the KASP assays predicted moderate CR haplotypes (allele 5). In contrast, array data suggested low (allele 3) and moderate (allele 5) haplotypes for ‘Peento’ and ‘Saturn’ and high (allele 6) and moderate (allele 5) haplotypes for ‘Fantasia’ (Table1; Supplementary Table S8).

**Table 3:** Primer info for Kompetitive allele-specific PCR (KASP).

Assay	Tm	Tag	Primer sequence (5' → 3')
Ppe.CR.1-1	54.1	F	*GAAGGTGACCAAGTTCATGCT*TTTGCACAAATTCCCATGAAAGAa
	54.5	H	*GAAGGTCGGAGTCAACGGATT*TTTGCACAAATTCCCATGAAAGAg
	56.4	N	GACCAAGAAATCAGCTTCAACTGTG
Ppe.CR.1-2	55.9	F	*GAAGGTGACCAAGTTCATGCT*TTCTCAAAAACCACTGCTTGCAt
	56.4	H	*GAAGGTCGGAGTCAACGGATT*TTCTCAAAAACCACTGCTTGCAg
	54.1	N	CCTCTGAAGAAGATTAACAAATATGTGAC
Ppe.CR.1-3	57.1	F	*GAAGGTGACCAAGTTCATGCT*GGTTCTCTTGGAAAGCTCCCTt
	57.5	H	*GAAGGTCGGAGTCAACGGATT*GGTTCTCTTGGAAAGCTCCCTc
	53.8	N	TCTGTAGTGGAGATATCATATTTCTCAC
Ppe.CR.1-4	54.4	F	*GAAGGTGACCAAGTTCATGCT*GGCTACTTTTGAATACTCTGGACt
	55.3	H	*GAAGGTCGGAGTCAACGGATT*GGCTACTTTTGAATACTCTGGACc
	56.3	N	CTGAGAATGCCCGTATAGGAGTAC

Annealing temperatures (Tm, °C) and primer sequences for each assay, including FAM (*GAAGGTGACCAAGTTCATGCT*) and HEX (*GAAGGTCGGAGTCAACGGATT*) tag at the 5' end of each forward primer. SNP nucleotide is shown in lower case in each forward primer sequence. ‘Tag’ indicates which fluorophore is used: ‘F’ = FAM, ‘H’ = HEX, ‘N’ = none, for the reverse primer.

From the 84 peach samples used for KASP assay validation, nine haplotype combinations (diplotypes) were reported (Table 1; Supplementary Table S8). In this material, six accessions had an L|L diplotype with a predicted phenotypic value lower than 350 CH, one accession had L|L-M (predicted CR < 450 CH), one L-M|L-M (predicted CR between 300 and 700 CH), four M|M-L (predicted CR between 400 and 750 CH), 33 M|M (predicted CR between 600 and 850 CH), two H|L-M (predicted CR between 500 and 850 CH), 23 H|M (predicted CR > 750 CH), and 14 accessions had H|H (predicted CR > 850 CH) diplotype (Table 1). Overall, KASP assays accurately predicted CH accumulation and actual CR phenotype in analyzed accessions (Table 4). The Ppe.CR.1 KASP assay had 74% accuracy in predicting the correct CR phenotype in the U.S. peach germplasm and selections (Tables 1 and 4), with only two diplotypes having accuracy below 70% (H|L-M, and M|M). For samples with H|L-M diplotype, a 50% accuracy was reported, although only two genotypes belonged to this group (Table 4; Supplementary Table S8). And for accessions with M|M diplotypes, a 42.4% accuracy was reported, with a higher number of discrepancies coming from accessions from A-pop. The KASP assays predicted moderate CR, and the actual CR observed in the forcing study was 350 CH (Table 1).

**Table 4:** Comparison between actual and predicted chill hour requirements obtained from Ppe.CR.1 assay in the U.S. germplasm and the Clemson University Peach Breeding (CUPB) program.

Diplotypes	Expected chilling hours	% Accuracy	
		Germplasm	CUPB
L | L	< 350	100 (6)	–
L | LM	< 450	100 (1)	–
L|M	< 650	–	–
LM | LM	300–700	100 (1)	83.3 (6)
M | LM	400–750	100 (4)	82.4 (17)
H | LM	500–850	50.0 (2)	71.4 (7)
M | M	600–850	42.4 (33)	70.0 (60)
H | M	> 750	87.0 (23)	85.9 (78)
H | H	> 850	71.4 (14)	83.3 (18)

Numbers in parenthesis indicate the number of individuals with this diplotype.

Even though all four KASP assays are needed to distinguish between the low, low-moderate, moderate, and high CR alleles, only one KASP assay, Ppe.CR.1-1, is needed to distinguish haplotypes lower and higher than 800 CH. All alleles with genotype ‘B’ for Ppe.CR1-1 showed less than 800 CH, whereas alleles with genotype ‘A’ for this assay had a mean CR higher than 800 CH (Table 2). Additionally, two Ppe.CR.1 assays, Ppe.CR.1-1 and -4, could distinguish between low (< 450 CH), moderate (450–800 CH), and high (> 800 CH) CR haplotypes (Table 2).

End-point PCR results agreed with the validation data, as the same four clusters (NTC, AA, AB, and BB) observed with real-time PCR runs were obtained (Supplementary Fig. S2).

### Seedling KASP genotyping

To test the efficiency of these assays (Ppe.CR.1-1 to -4), we screened 161 seedlings from 63 crosses of the CUPB program and 25 peach cultivars using a rapid DNA extraction protocol (Supplementary Table S1). CR of this plant material was obtained in 2021/2022 by bud break forcing, and raw fluorescence data from the end-point PCRs were used for SNP genotype assignment.

The KASP genotyping revealed six out of seven haplotypes found in the U.S. germplasm in this material, except low CR allele 3 (Supplementary Table S1). Additionally, haplotype combinations resulted in six different diplotypes, with the number of individuals ranging from six (LM | LM) to 78 (H | M) (Table 4). Similarly, to the validation assays, high efficiency in CR prediction (79.6% of accuracy) by Ppe.CR.1 assays was observed in this breeding material. A comparison of predicted and observed CR phenotypes for the CUPB material revealed an accuracy higher than 70% for all diplotype combinations identified (Table 4).

## Discussion

Marker-assisted breeding relies on identifying a DNA pattern associated with a trait^40,41^. Identifying an association between a trait and a marker allows breeders to screen a population through genetic testing. It enables a more streamlined breeding pipeline letting breeders distinguish between desired and undesired germplasm before growing a plant to maturity. Countless DNA markers are associated with traits such as fruit quality, disease resistance, maturity date, and postharvest quality in peach^2,26,42^. However, very few have been converted into diagnostic DNA tests for breeders^43^. Most of the studies underlying the genetics behind relevant breeding traits stop at the discovery of the QTLs or associations and never go the extra step to develop DNA diagnostic tools(s) for breeders to apply in their programs. The complexity of trait genetics makes it difficult to develop and validate DNA markers for some traits. For example, traits with a simple genetic basis, such as bacterial spot resistance^33^, skin blush coverage^43^, flesh color^44^, or peach-nectarine type^45^ are ideal for DNA test development. For these traits, the largest percentage of phenotypic variation is controlled by one or two major genes. However, for most of the quantitative traits, in which phenotypic variation is controlled by many genes with small effects, a DNA test is not an efficient tool, so breeders need to use genomic prediction to account for all the genes associated with the trait and therefore predict the phenotype. In addition, validation is required to make sure the DNA test predict the actual phenotype and is applicable to various background. Thus, despite numerous markers associated with fruit quality, postharvest, or disease resistance are discovered, few of them get translated into diagnostic DNA tests.

Few DNA tests are available for peach, and most are associated with fruit quality^43^ or disease resistance^33^. Only one attempt to develop a DNA test to predict CR in peach was reported^34^. However, insufficient information was published, preventing replication of the key test. Additionally, the authors used HRM analysis, which is not user-friendly to many breeders, and the DNA toolkit was validated in a few cultivars, mostly from Asian germplasm^34^. Therefore, we set out to develop the Ppe.CR.1 DNA test using the KASP approach for the major chill-associated region in peach on the bottom of Chr1 (42.5–44.0 Mbps) that overlaps with the DAM genes in peach (Chr1: 43.41–43.48 Mbps), which are considered the major genes associated with the phenotypic variation of QTLs in this region^16,24^. Using allelic positions from previously reported QTLs on LG1^14–19^ and our efforts^35^, we narrowed down the region of interest and selected SNPs for KASP assay development. The developed KASP assays successfully predicted CR genotype (96.6% accuracy) and CR phenotype (74% accuracy) in the U.S. breeding germplasm. Additionally, a higher accuracy (80%) in predicting the CR phenotype was achieved in selections from the CUPB program. The CR of the CUPB material was determined by forcing bloom after a known chill hour exposure, while the CR of the material used for validation was acquired from the literature. CR phenotype of the peach germplasm reported in the literature was obtained either by greenhouse forcing or simply by comparing bloom dates and assigning the CR of a cultivar with a similar bloom date^37^. The discrepancy in CR data quality might be responsible for lower accuracy in predicting the CR phenotypes in the validation material. Additionally, in this study, we did not account for the interaction between the three major CR QTL regions on Chrs 1, 4, and 7 in the peach genome^14–19^. We focused on the CR QTL region on Chr1 for developing DNA test(s) because of its most significant effect on CR phenotype in peach germplasm observed across all studies (> 50% of phenotypic variance explained). Thus, the prediction accuracy of the Ppe.CR.1 assays could also be affected by a potential epistatic effect between these three CR regions. Still, more than 70% accuracy in predicting CR phenotype was achieved, making the Ppe.CR.1 a quick and accurate tool to use in peach breeding programs.

During the development and validation of this assay, both real-time and end-point PCR results were in agreement, revealing that adopting end-point PCR with crude DNA extraction in a routine application can increase the throughput of this breeding tool after optimizing reaction parameters with real-time PCR^33^. Accuracy of the Ppe.CR.1 KASP assays in predicting SNP genotypes and assembly of correct haplotype in the validation step was checked using germplasm that was already genotyped with peach 9K SNP array v1^21,46,47^. In our previous study, we already assembled the haplotypes of these accessions and predicted the CR phenotype using the array data^35^. KASP and 9K SNP array v1 genotypes disagreed for only three samples (‘Fantasia’, ‘Saturn’, and ‘Peento’). A possible explanation for this discrepancy is that array genotyping results were not correctly called or that the source of DNA used in the RosBREED genotyping project^48,49^ and in the validation stage in this study was not the same (true to type). For example, discrepancies were observed for ‘Peento’ and ‘Saturn’ cultivars, and these names are often used to refer to flat peaches in general. The 9K SNP array has been widely used in peach breeding programs for genotyping important breeding parents and most peach breeders already have genotypic data for their most important material. However, the peach array has been decommissioned and is no longer available for genotyping new material. Therefore, the high accuracy of the Ppe.CR.1 in matching array genotypes allows breeders to use existing parental array genotypic information to design crosses and evaluate progeny.

Our research group recently reported haplotypes for the major CR-related region on LG1 of a large peach germplasm relevant for the U.S. peach breeding programs^35^. This information provided relevant data for cross-design in breeding programs using known cultivars to plan crosses to achieve CR breeding goals. That work set the foundation for the development of the Ppe.CR.1 KASP assays that allow the prediction of CR phenotype in unknown germplasm and selecting desirable genotypes in seedlings in the early development stages. The KASP assay (Ppe.CR.1) presented here has several advantages over the previously reported CR DNA toolkit^34^. Chou et al.^34^ DNA assays are based on HRM techniques and require specialized equipment and high-quality DNA that are not always available nor affordable to breeders. The Ppe.CR1 only requires real-time PCR equipment for reading the fluorescent signal and can be run on end-point PCR equipment with crude DNA extract^33,36^. We have also employed the delta method developed by Fleming et al.^33^ to quickly adjust genotype assignments according to the controls and correctly assign SNP calls. This approach was adjusted for Ppe.CR.1 KASP assays and is freely available. Additionally, a lower number of markers are needed for CR prediction with Ppe.CR.1. The HRM toolkit developed by Chou et al.^34^ uses 22 CR-associated markers for SNP genotyping and R script for PCA variant calling pipeline for CR prediction and is relevant to the Taiwan and Southeast Asia germplasm. In contrast, four Ppe.CR.1 assays are capable to distinguish between low, low-moderate, moderate, and high CR haplotypes/phenotypes, and is validated in the U.S. peach breeding germplasm, which shares pedigree with most of the European peach germplasm^21^, making this test applicable for predicting CR in most peach breeding programs.

Considering observed genotypes for each Ppe.CR.1 allele/haplotype and corresponding phenotype our recommendation is to apply step approach and first use the Ppe.CR.1-1 (SNP_IGA_134730) assay. This assay distinguishes between chilling requirement lower and higher than 800 CH, as individuals with genotype ‘B’ need less than 800 CH, whereas those with ‘A’ require more than 800 CH. This simple test provides the tool to remove seedlings that require too much chilling if breeding for less than 800 CH is the target, such as the case in the CUPB. In the second step, breeders can use Ppe.CR.1-4 assay to further determine between low, moderate, and high CR alleles. Individuals with genotype ‘A’ for Ppe.CR1-4 are associated with CH reduction and genotype ‘B’ with increased chilling accumulation. Therefore, for these two assays, Ppe.CR1-1 and -4, individuals homozygous for ‘A’ (A|A) suggest high CR (~ 800 CH), homozygous for ‘B’ (B|B) moderate CR (600–700 CH) and heterozygous ‘B|A’, respectively, low CR (< 500 CH) (Table 2). With this step approach funding required for seedling selection based on the CR could be optimized.

## Conclusion

This study provides a quick, reliable, and user-friendly DNA-based toolkit with optimized primers for assessing the genotypes of four CR-related SNP markers. KASP assays for CR-related SNPs were developed and validated on individuals with known and unknown genotypes. These assays were highly accurate, identifying the correct genotype for 97% of samples with known genotypes. The high predictive accuracy of the KASP assays and the ability to distinguish between low and high CR using only a single KASP assay provides a quick and affordable test to eliminate the material that has high CR in programs breeding for low CR and vice versa. In addition, using only two KASP assays, we can predict CR phenotypes for individuals lower than 400, between 400 and 800, and more than 800 CH and select the targeted CR class the breeding program aims for. The KASP approach reported here provides efficient, low-cost analyses that supply relevant information to breeders for culling undesirable seedlings before field evaluation. The efficiency of KASP assays coupled with low-cost crude DNA extract enables a marker-assisted selection approach for large-scale seedling selection for CR in the peach breeding programs.

## Supplementary Information

Supplementary Information 1.

Supplementary Information 2.

## Data Availability

All datasets generated during the current study are available in tables, figures, and supplementary information and Genome Database for Rosaceae (https://www.rosaceae.org; accession number: tfGDR1048/b).
