# Supplementary material for: Ppe.CR.1 DNA test for predicting chilling requirement in peach
Source: Sci Rep. 2023 Jan 18;13:987. doi: 10.1038/s41598-023-27475-w (PMC9849201; doi:10.1038/s41598-023-27475-w)

Ppe.CR.1 DNA test for predicting chilling requirement in peach

Gizem Demirel[1,2,+], Alejandro Calle[1,+], John Mark Lawton[1], Omer Atagul[1,3], Wanfang Fu[1] and Ksenija Gasic[1,*]

[1]Department of Plant & Environmental Sciences, Clemson University, Clemson, SC 29634, USA.
[2]Department of Breeding and Genetics, Fruit Research Institute, 32500 Isparta, Turkey.
[3]East Mediterranean Transitional Zone Agricultural Research Institute, 46060 Kahramanmaras, Turkey.

[*]Corresponding author: kgasic@clemson.edu
[+]these authors contributed equally to this work

Supplementary Figure 1. Positions of previously reported quantitative trait loci (QTLs) for chilling requirement (red) and bloom date (black) in chromosome 1 of the peach genome (Fan et al. 2010; Romeu et al. 2014; Zhebentyayeva et al. 2014; Bielenberg et al. 2015; Cantín et al. 2020; Rawandoozi et al. 2021). The scale indicates the physical position (Mbp). The orange square region indicates the interval considered for haplotype construction.

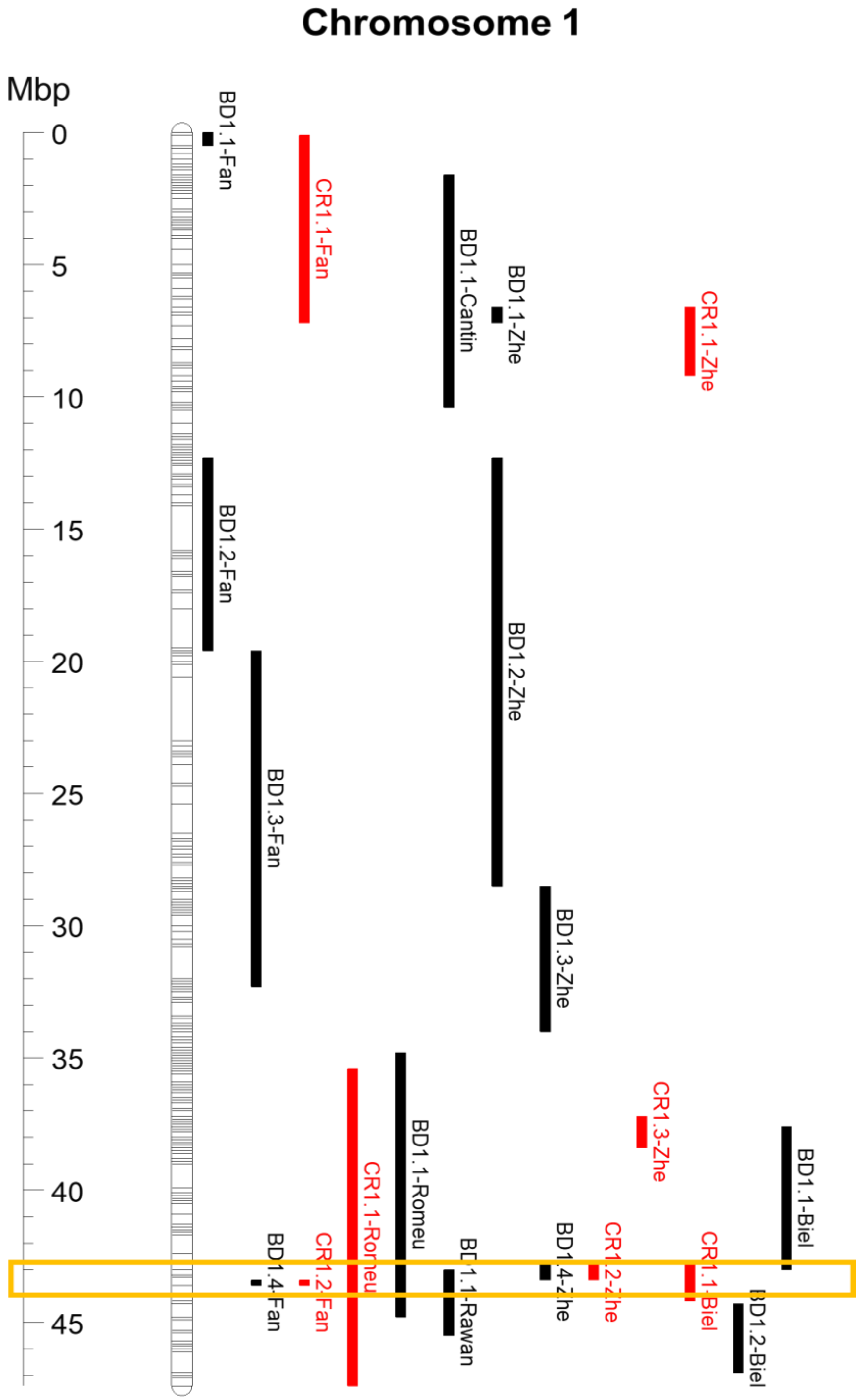

Supplementary Figure 2. Comparison of FAM and HEX fluorescence values for each KASP assay using real-time (A) and endpoint (B) PCRs in the validation plate. Circles, triangles, diamonds, and squares indicate no amplification, AA, AB, and BB genotypes, correspondently.

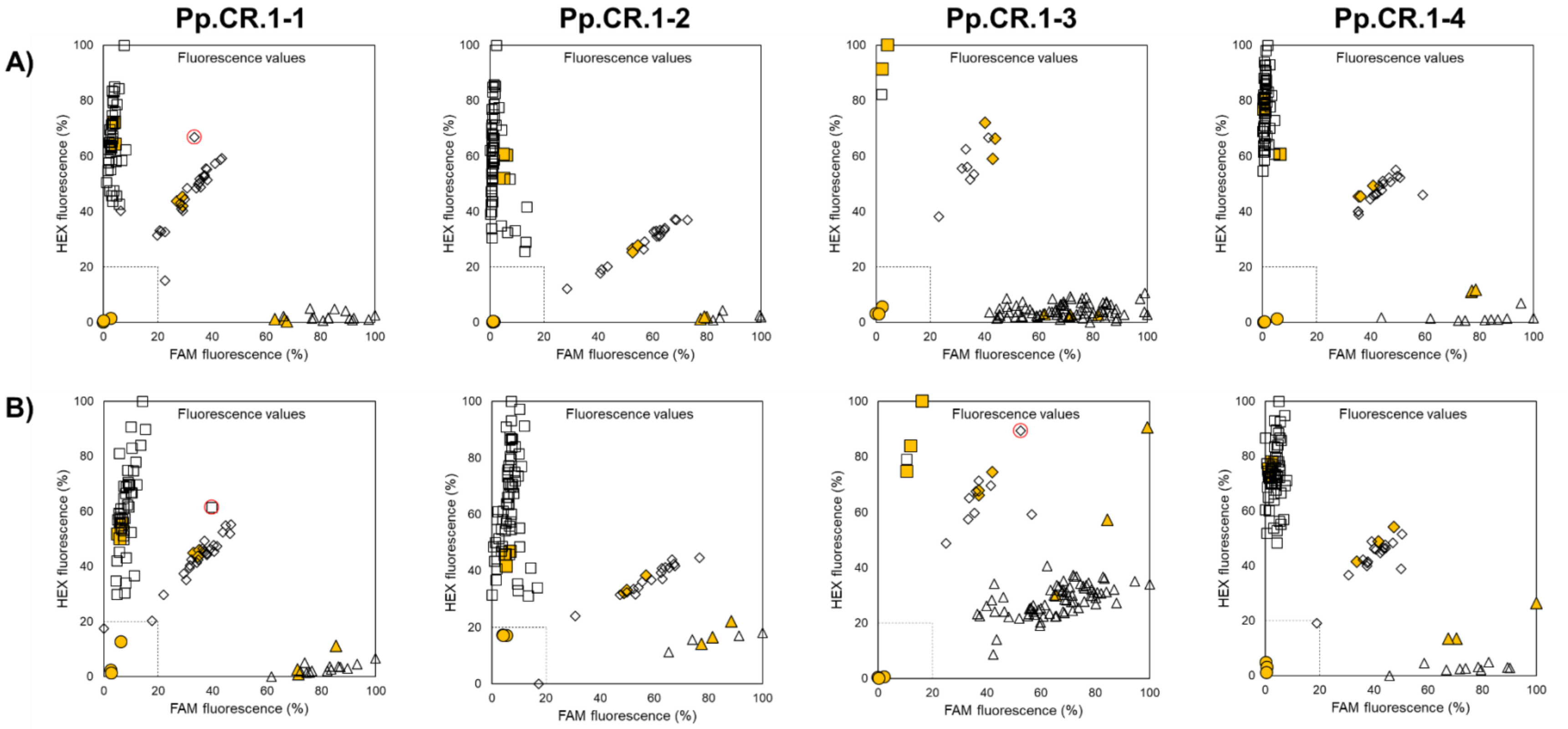

Supplement: Supplementary file 1 — Supplementary Information 1. [file 41598_2023_27475_MOESM1_ESM.pdf]
